# Chromatographic Characterization and GC-MS Evaluation of the Bioactive Constituents with Antimicrobial Potential from the Pigmented Ink of *Loligo duvauceli*

**DOI:** 10.1155/2014/820745

**Published:** 2014-11-10

**Authors:** Smiline Girija, Veeramuthu Duraipandiyan, Pandi Suba Kuppusamy, Hariprasad Gajendran, Raghuraman Rajagopal

**Affiliations:** ^1^Department of Microbiology, Meenakshi Ammal Dental College and Hospital, Meenakshi Academy of Higher Education and Research (MAHER), Meenakshi University, Madhuravoyal, Chennai, Tamilnadu 600 095, India; ^2^Entomology Research Institute, Loyola College, Chennai 34, Tamilnadu, India; ^3^Department of Microbiology, Meenakshi Ammal Dental College and Hospital, Meenakshi University, Madhuravoyal, Chennai, Tamilnadu 600 095, India; ^4^Department of Microbiology, Government Thoothukudi Medical College, Thoothukudi, Tamilnadu, India

## Abstract

Chromatographic characterization and the GC-MS evaluation of the black pigmented ink of *Loligo duvauceli* in the present study have yielded an array of bioactive compounds with potent antimicrobial property. Facing an alarm of antimicrobial resistance globally, a need for elucidating antimicrobial agents from natural sources will be the need for the hour. In this view, this study is aimed at characterizing the black pigmented ink of the Indian squid *L. duvauceli*. The squid ink was subjected to crude solvent extraction and was fractionated by silica gel column chromatography. TLC and HPTLC profiles were recorded. Antimicrobial bioassay of the squid ink fractions was done by agar well diffusion method. The antimicrobial fraction was then characterized using GC-MS analysis. The results showed that the *n*-hexane extract upon column fractionation yielded a total of 8 fractions with the mobile phase of Hex/EtOAc in different gradients. TLC and HPTLC profiles showed a single spot with a retention factor of 0.76. Fraction 1 showed significant antibacterial activity against *Escherichia coli*, *Klebsiella pneumoniae*, *Staphylococcus aureus*, and *Lactobacillus acidophilus* and a promising antifungal activity against *Candida albicans*. The antimicrobial fraction upon GC-MS analysis of bis(2-ethylhexyl) phthalate (BEHP) possesses the highest percentage of area normalisation (91%) with other few minor constituents. The study is concluded by stating that the antimicrobial efficacy of the squid ink might be due to the synergistic effects of the phthalate derivative and the other minor volatile compounds analysed in the squid ink.

## 1. Introduction

Characterization of the bioactive constituents from the black pigmented ink has resulted in a handful of chemical elucidations. The ink from the molluscs has created a great interest towards its bioactive molecules with promising antibacterial, antitumour, antileukemic, and antiviral activities [1]. The ink is ejected from the ink gland of the squid* Loligo duvauceli *through the ink duct to escape from its predators [2]. High performance liquid chromatographic (HPLC) analysis of the* Loligo *sp. ink has quantified its chemical components as L-DOPA and Dopamine [3]. The black pigment was found to be melanin and the process of melanogenesis was explained in the ink gland of* Sepia* sp. [4]. The ink is a complex mixture of organelles, premelanosomes, melanosomes, granules, proteic material (enzymes), glucosamine, and phospholipids in suspension. At the moment of extraction the mixture is still active which makes the ink suitable for research studies. The ink gland has been also shown to contain a variety of melanogenic enzymes as tyrosinase, dopachrome tautomerase, and peroxidase [5]. The ink has also various primary roles in the world of alternative medicine and has the widest range of therapeutic application [6].

Despite these reports there is no considerable interest shown towards the purification procedures of the* L. duvauceli*'s ink. Potential chemical cues in squid ink have been identified and quantified using reverse-phase, high-performance, liquid chromatography (RP-HPLC) [7]. Meanwhile the active antimicrobial biomolecules have not been characterized yet. So this study is aimed at exploring the active bioconstituents of the ink by silica gel column chromatography and the gas chromatography-mass spectroscopic analysis (GC-MS) for its antimicrobial constituents.

## 2. Materials and Methods

### 2.1. Preparation of Crude Extracts

The collection of ink and the crude solvent extraction of the constituents from* L. duvauceli *ink was done by the method followed earlier [8]. The crude extracts were subjected to sterility checking after exposing the extracts under UV light for 2 hrs. 5 mg of each extract was mixed in sterile nutrient broth and was incubated for 2 hrs which was plated onto nutrient agar for checking the sterility of the extracts. The extracts were stored at 4°C in brown glass bottles. The antimicrobial activity of the crude extracts was performed by conventional agar well diffusion method [9]. The* n-*hexane extract has scored in our earlier reports a high antimicrobial property against the clinical bacterial and fungal isolates [10]. Thus the* n-*hexane extract was chosen for the further fractionation by silica gel column chromatography.

### 2.2. Chromatographic Fractionation of the Hexane Extract

Separation of the active biomolecules from the crude* n-*hexane extract was done by silica gel column chromatography. Briefly, 10 gm of the crude* n-*hexane extract was subjected for fractionation using silica gel column. The crude extract was adsorbed on to silica gel (100–200 mesh, SISCO) and chromatographed employing a step gradient solvent system from low to high polarity. The starting solvent system was 100%* n-*hexane and subsequently the polarity was increased by varying the solvent concentration with ethyl acetate (EtOAc). The solvent gradient for the chromatogram is 100% hexane, 20 : 80 EtOAc/Hex, 40 : 60 EtOAc/Hex, 60 : 40 EtOAc/Hex, 80 : 20 EtOAc/Hex, 100% EtOAc, 100% diethyl ether, 20 : 80 Ethanol/EtOAc, 40 : 60 Ethanol/EtOAc, 60 : 40 Ethanol/EtOAc, 80 : 20 Ethanol/EtOAc, 100% Ethanol, 20 : 80 Water/EtOAc, 50 : 50 Water/EtOAc, and 100% Water. In order to select the best mobile phase for eluting the fractions, 5 *μ*L of each eluted fraction was spotted on TLC and ran with combinations of solvent system. In this way the solvent system that showed the most favorable separation of compounds was chosen. The fractions that showed the elution of similar compounds were pooled and concentrated under vacuum below 40°C using Heidolph, VE-11 Rotaevaporator for 30 min. High performance thin layer chromatography (HPTLC) analysis was also performed for the active fraction. Combined fractions were kept under air current to facilitate drying. The concentrated fraction was obtained and subjected to sterility checking as mentioned earlier. The active fraction was stored at 4°C in sterile glass brown bottles until used for the bioactivity studies.

### 2.3. Antimicrobial Bioassay

The antimicrobial activity of isolated fractions was checked against* Escherichia coli* (ATCC 25922),* Klebsiella pneumoniae* (ATCC 10031),* Staphylococcus aureus* (ATCC 25923),* Candida albicans* (ATCC 10231), and* Lactobacillus acidophilus* [MTCC 447]. The bioassay was performed by agar well diffusion method. Briefly, Mueller Hinton agar plate was divided into two halves and 50 *μ*L of inoculum of each test organism was spread as lawn cultures on the same plate to achieve a confluent growth at each half. The agar plates were allowed to dry and wells or cups of 8 mm were made with a sterile agar borer on the inoculated agar plates. 10 mgs of the pooled fraction was mixed with DMSO and was made ready for the study. A 50 *μ*L volume of the active fraction was propelled directly into the wells of the inoculated specific media agar plates for each test and control organism. Erythromycin (30 *μ*g) and Amphotericin B (100 U) were used as the positive controls for the bacteria and the yeast, respectively. DMSO served as the negative control. The plates were allowed to stand for 10 minutes for diffusion of the extract to take place and were incubated at 37°C for 24 h. After incubation the plates were observed for the zone of inhibition around the wells and the zone of inhibition was measured using an antibiotic sensitivity measuring scale (Himedia, Mumbai).

### 2.4. Determination of MIC and MBC Value for the Active Fraction

Determination of MIC value for the active antimicrobial fraction was determined by Microbroth dilution method [11]. Serial dilutions of the active fraction were done in a 96-well microtitre plate with DMSO. The dilution factor was 5, 2.5, 1.25, 0.625, 0.312, and 0.156 mg/mL. To each dilution 100 *μ*L of the culture broths of the test organisms was added in their respective wells and the plate was incubated at 37°C for 24 hrs. After incubation the spectrophotometric analysis was performed and the OD values were recorded. The MBC value was confirmed by microbial spot checker board method [12] where 3 *μ*L of each dilution was spotted onto Mueller Hinton agar plates and incubated at 37°C for 24 hrs. After incubation the spot showing the complete absence of microbial growth indicates the minimum bactericidal value and the spot showing the visible decrease in the number of colonies indicates the minimum inhibitory concentration (MIC value).

### 2.5. GC-MS Analysis of the Active Fraction

The active fraction was then subjected to GC-MS analysis with the split injection ratio of 1 : 20 in hexane in Shimadzu GC-MS QP 2010 (Japan) gas chromatograph with capillary column length of 30 m, 0.25 mm diameter, and 0.25 *μ*m of film thickness. GC-MS operating conditions were as follows: injector temperature at 280°, column oven temperature at 45°, flow control mode at linear velocity, and column flow of helium (99.9% purity) at 1.40 mL/min. Oven temperature programme was maintained at 45°C for 2 min and 300°C for 10 min with overall holding time of 36.5 min. Mass spectra conditions applied were as follows: electron impact at 40 eV, ion source temperature at 200°C, and interface temperature at 240°C. Individual components were identified by Wiley 139.LIB and NISTO.5 LIB database matching. The percentage composition was determined by area normalization.

## 3. Results

**Figure 1:**
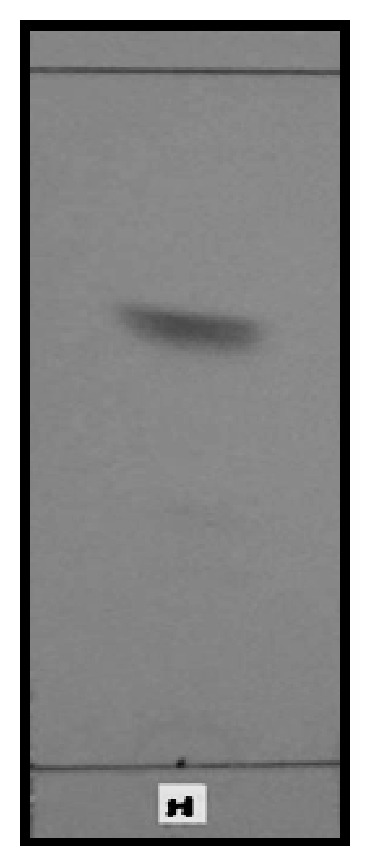
TLC profile of the active fraction [developing system:* n-*hexane : ethyl acetate (4 : 1), visualisation: UV −254 nm].

The* n-*hexane extract scoring a high antimicrobial activity upon column fractionation over silica gel yielded a total of 8 fractions. Elution with Hex/EtOAc in the ratio of 4 : 1 yielded the active fraction. The active fraction was subjected to TLC, HPTLC, and GC-MS analysis. TLC profile showed a single spot with a retention factor of 0.76 (Figure 1). The same plate was subjected to HPTLC analysis, with a scanning wavelength of 254 nm. The chromatogram showed a single peak obtained as calibration spectrum data with a noise level at 0.072 mV, CAMAG software, and scanned with SCANNER II [951012] with area normalization of 83.91% that indicated the maximum extraction with Hex/EtOAc.

**Figure 2:**
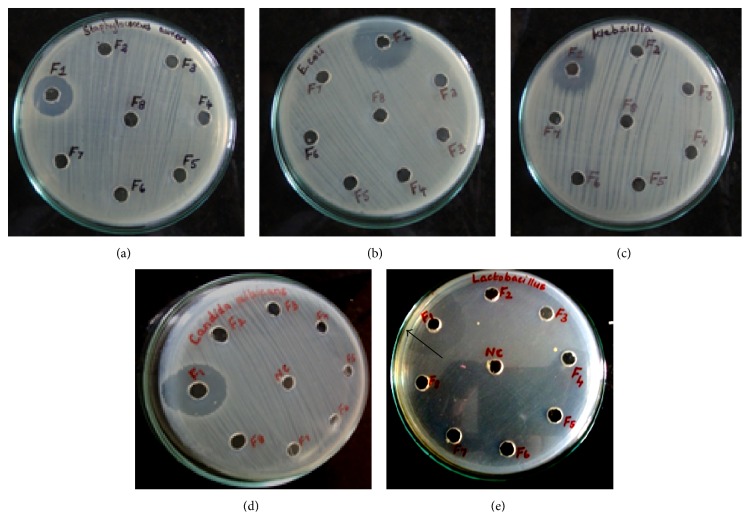
Antimicrobial activity of Fraction 1 showing the zone of clearance against (a)* S. aureus*, (b)* E. coli*, (c)* K. pneumoniae*, (d)* L. acidophilus*, and (e)* C. albicans*.

**Figure 3:**
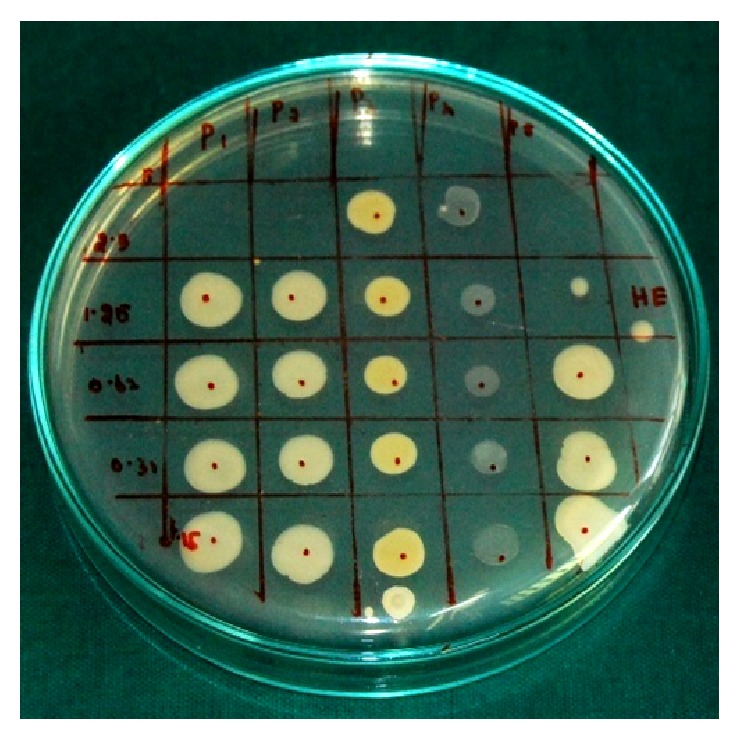
Microbial spot checker board assay.

Antimicrobial bioassay revealed that the active fraction possesses a high antibacterial activity against the test organisms (Table 1). The zone size was measured as 18 mm for* E. coli* and* K. pneumoniae*, 16 mm for* S. aureus*, 23 mm for* C. albicans*, and 18 mm for* L. acidophilus* (Figure 2). The MBC value was determined as an average of 2.5 mg/mL for* E. coli*,* K. pneumoniae*, and* C. albicans*, 5 mg/mL for* S. aureus* and* L. acidophilus*. The microbial spot checker (Figure 3) board method yielded complete absence of the growth at the spot inoculated with the determined MBC value. The previous dilution that showed the visible decrease in the number of colonies was determined as the MIC and was deduced as 1.25 mg/mL for* E. coli*,* K. pneumoniae*, and* C. albicans* and 2.5 mg/mL for* S. aureus* and* L. acidophilus*.

**Figure 4:**
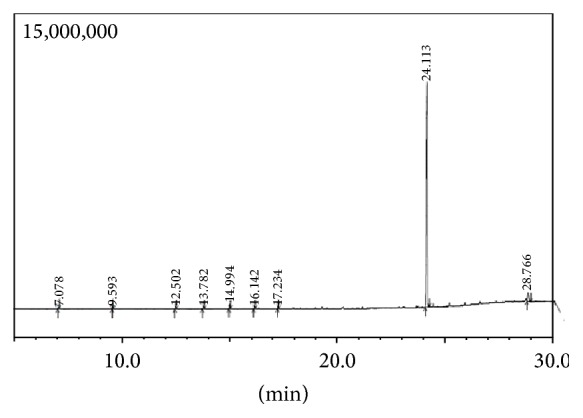
GC-MS chromatogram of the active fraction.

The bioactive fraction upon GC-MS analysis revealed a chromatogram showing nine peaks with bis(2-ethylhexyl) phthalate [BEHP] possessing the highest percentage of area normalisation (91%) (Figure 4). The mass spectrum was found to be superimposable (>93) with that of the authentic compound from the GC-MS library. Based on the GC-MS analysis the active fraction was structurally elucidated as bis(2-ethylhexyl) phthalate. The chromatogram also showed the presence of other minor compounds such as octadecane (0.29%), naphthalene (0.13%), tetradecane (0.41%), pentadecane (0.58%), hexadecane (1.02%), heptadecane (0.53%), and cholesterol (5.07%) (Table 2). The analysis report reveals the presence of phthalate derivative and other minor volatile essential oils as potent antimicrobial agents extracted from the squid ink.

## 4. Discussion

Marine natural products have been a strong source for novel drug products, or have been a model for introducing a commercial drug [13]. Squid ink is not the most elusive and enigmatic pigment found in nature but just a particle waiting for a rational study. In recent years, the problem of antimicrobial (drug) resistance is emerging and many diseases are increasingly difficult to treat because of the emerging drug-resistant organisms [14]. The design of effective and novel dosing regimens that suppress the emergence and proliferation of resistant microbial populations is crucial [15]. As resistance has increased to alarming proportion, a safe and cheaper source can always be an alternative to the routine therapeutics. As mollusk has been reported to possess various active molecules, the ink from the squid* L. duvauceli* is selected as a novel source for the isolation of antimicrobial agents.

Previous associated studies state that the crude extraction of the squid ink is achieved using various solvents [16]. Successful prediction of natural bioactive molecules from natural sources is largely dependent on the type of solvent used in the extraction procedure and in many studies it was found that extracts in organic solvents provided more consistent antimicrobial activity [17]. The crude extraction in our earlier studies is thus achieved by parallel solvent extraction method, where the ink is mixed with various solvents individually and is not added sequentially. Separation of the biomolecules has been successfully achieved by chromatographic procedures. TLC and HPTLC analysis are the simplest and cheapest method of detecting any natural constituent because the method is easy to run and reproducible and requires little equipment. The Rf value correlates with the phthalate compound isolated from the other sources [18]. Active crude extracts are chosen for column chromatography due to its relatively low complexity as seen with TLC, bioautography, and disc diffusion method [19]. The solvent gradient used for elution of the biomolecules has been successfully standardized and is best achieved with* n-*hexane and ethyl acetate gradient. The selection of the solvent gradients can be rationalized in terms of the polarity of the compounds being extracted by each solvent and in addition to their intrinsic bioactivity, by their ability to dissolve or diffuse in the different media used in the assay [20].

For the antimicrobial bioassay, the concentrated fractions were dissolved with DMSO and were employed for the agar well diffusion bioassay. The choice of DMSO as a solvent is due to its solvency for a wide range of chemicals, its low antibacterial activity at concentrations less than 2%, and its low toxicity [21]. The findings of the antimicrobial bioassay report that the active fraction has antimicrobial efficacy against the Gram positive cocci* S. aureus*, Gram negative bacilli* E. coli* and* K. pneumoniae*, Gram positive bacilli* L. acidophilus*, and the pathogenic yeast* C. albicans*. The active fraction scores a high activity against* C. albicans* that indicates its potent antifungal activity. A good antibacterial activity is achieved against the Gram negative and positive bacilli. A moderate antibacterial activity is observed against* S. aureus*. The MBC value has also been deduced and is determined as 2.5 to 5 mg/mL against the tested organisms. Microbial spot checker board also yields the same result with the absence of growth at the spot inoculated with the determined MBC value.

GC-MS analysis of the bioactive fraction shows the presence of the bioactive compounds which are further confirmed with the library data. Using mass spectroscopy the molecular mass of a compound and its elemental composition can be easily determined. Further this method involves very little amount of the test sample and gives the molecular weights accurately. GC-MS analysis has showed bis(2-ethylhexyl) phthalate as the major constituent with large area normalization of 91.43%. BEHP identified through this analysis has been further confirmed by its molecular mass spectrum. It correlates with the mass spectrum of BEHP reported earlier [22] from a phthalate isolated from a marine bacterial strain. The other minor constituents with low area normalization were also identified by the molecular mass from the library database.

Bis(2-ethylhexyl) phthalate is found in low levels in the environment as it is subjected to biodegradation [23]. This derivative has also been reported to be present in the fish and lipid tissues and also as a pollutant in marine environment [24]. The bioactivity of the phthalate derivatives has already been reported in many plants, algae, and marine microorganisms and also from many marine species [25]. Few reports are available for the antibacterial potential of phthalate derivatives from plants and from flowers [26]. Bis(2-ethylhexyl) phthalate extracted from* Streptomyces bangladheshiensis* has been reported to be a potent antibacterial agent against Gram positive bacteria [27]. Di(2-ethylhexyl) phthalate from* Alchornea* sp. has proved to reduce anti-inflammatory activity [28]. The other volatile minor compounds identified by GC-MS analysis have also been found to be potent antibacterial agents. The extracts of* Spirulina* sp. have showed antibacterial activity of octadecane and tetradecane [29]. The antibacterial activities of pentadecane and heptadecane compounds extracted from Sea Urchin have also been reported to possess potent activity against Gram positive and Gram negative bacteria [30]. BEHP has been reported to possess a potent antifungal activity against major pathogenic fungi like* Candida*,* Cryptococcus*, and* Aspergillus* sp. [31]. The other minor constituents analyzed by GC-MS have also been reported to possess antifungal activity. Naphthalene derivative has been reported to possess the same against* C. albicans *and* Aspergillus* sp. [32]. The antifungal activity of cholesterol hydrazone derivative has also been studied against* C. albicans* at a concentration of 1.5 *μ*g/mL [33]. The antifungal activity of tetradecane and octadecane has been reported against* C. albicans* [34]. GC-MS analysis of a natural cure concoction Epa-Ijebu showed the presence of natural alkanes such as hexadecane, heptadecane, and octadecane with potent antifungal activity [35]. In correlation with these reports, the study results reveal that the ink has potent antimicrobial constituents which owes for its antibacterial and antifungal properties. The promising antimicrobial activity of these bioactive constituents needs a further multipronged research to implement its use as a novel therapeutic agent in near future for treating ailments with the drug resistant microbial pathogens.

This study has suggested the presence of antimicrobial bioconstituents in the squid ink by column fractionation studies. GC-MS analysis has also aided the evaluation of the major and minor compounds present in it through its mass spectrum data. The study thus concludes the synergistic effects of an array of compounds in the squid ink towards its potent antimicrobial property. A novel therapeutic compound from a new marine source like the squid ink would be of much use in eradicating the microbial pathogens and it would definitely aid in the control and emergence of drug resistant strains.

## Figures and Tables

**Table 1:** Column fraction 1 showing a prominent zone of clearance [in mm] against the test organisms.

Organisms under study	Zone of inhibition for the tested fractions (in mm)								MIC value (in mg/mL) for active fraction 1
	1	2	3	4	5	6	7	8	
*Escherichia coli* (ATCC 25922)	18	—	—	—	—	—	—	—	2.5
*Klebsiella pneumoniae* (ATCC 10031)	18	—	—	—	—	—	—	—	2.5
*Staphylococcus aureus* (ATCC 25923)	16	—	—	—	—	—	—	—	5
*Candida albicans* (ATCC 10231)	23	—	—	—	—	—	—	—	2.5
*Lactobacillus acidophilus* (MTCC 447)	18	—	—	—	—	—	—	—	5

—: no activity; 1–8: isolated column fractions.

**Table 2:** GC-MS analysis of active antimicrobial fraction [fraction 1] from the ink of *L. duvauceli* revealing the bioactive constituents.

S. number	Retention time	Area %	Compounds
1	7.078	0.29	Octadecane
2	9.593	0.13	Naphthalene
3	12.502	0.41	Tetradecane
4	13.782	0.58	Pentadecane
5	14.994	1.02	Hexadecane
6	16.142	0.53	Heptadecane
7	17.234	0.54	Octadecane
8	24.113	91.43	Bis(2-ethylhexyl) phthalate
9	28.766	5.07	Cholesterol
